# The Role of Human Transportation Networks in Mediating the Genetic Structure of Seasonal Influenza in the United States

**DOI:** 10.1371/journal.ppat.1004898

**Published:** 2015-06-18

**Authors:** Brooke A. Bozick, Leslie A. Real

**Affiliations:** 1 Population Biology, Ecology, and Evolution Program, Emory University, Atlanta, Georgia, United States of America; 2 Center for Disease Ecology, Emory University, Atlanta, Georgia, United States of America; 3 Department of Biology, Emory University, Atlanta, Georgia, United States of America; Cambridge University, UNITED KINGDOM

## Abstract

Recent studies have demonstrated the importance of accounting for human mobility networks when modeling epidemics in order to accurately predict spatial dynamics. However, little is known about the impact these movement networks have on the genetic structure of pathogen populations and whether these effects are scale-dependent. We investigated how human movement along the aviation and commuter networks contributed to intra-seasonal genetic structure of influenza A epidemics in the continental United States using spatially-referenced hemagglutinin nucleotide sequences collected from 2003–2013 for both the H3N2 and H1N1 subtypes. Comparative analysis of these transportation networks revealed that the commuter network is highly spatially-organized and more heavily traveled than the aviation network, which instead is characterized by high connectivity between all state pairs. We found that genetic distance between sequences often correlated with distance based on interstate commuter network connectivity for the H1N1 subtype, and that this correlation was not as prevalent when geographic distance or aviation network connectivity distance was assessed against genetic distance. However, these patterns were not as apparent for the H3N2 subtype at the scale of the continental United States. Finally, although sequences were spatially referenced at the level of the US state of collection, a community analysis based on county to county commuter connections revealed that commuting communities did not consistently align with state geographic boundaries, emphasizing the need for the greater availability of more specific sequence location data. Our results highlight the importance of utilizing host movement data in characterizing the underlying genetic structure of pathogen populations and demonstrate a need for a greater understanding of the differential effects of host movement networks on pathogen transmission at various spatial scales.

## Author Summary

The rapid, long-distance spread of human pathogens such as seasonal influenza A across modern transportation networks presents a tremendous challenge for public health. Previous work based on influenza-like illness reports has demonstrated that commuters play an important role in the transmission of influenza across the United States. However, genetic structuring of influenza populations within a single season has not previously been detected. Here, we use sequence data collected over multiple seasons to investigate how human movement along the aviation and commuter networks in the United States contributes to influenza transmission at the regional scale. We confirm that commuters can play an integral role in interstate influenza transmission, but found that this pattern was specific to the influenza A subtype under investigation. We additionally show that strong county-to-county commuter flows do not necessarily fall within state boundaries, emphasizing the need for more precise spatial data to be associated with publically available sequences. Our results demonstrate that genetic structure does exist for influenza populations during the course of a single season at the regional scale and highlight the need to incorporate host movement patterns when studying spatial population structure.

## Introduction

When infectious agents invade naïve host populations and are propagated predominantly by local transmission, we expect to observe wave-like spread across geographic space [1–3]. Local transmission processes should concomitantly generate patterns of pathogen genetic variation approximating isolation-by-distance, where the geographic distance between locations and the genetic distance between pathogen variants is positively correlated [4, 5]. However, for pathogens of humans and other hosts that frequently travel long distances or along pathways not determined by local geography (e.g. aviation networks), accounting for species-specific movement patterns provides an alternative method of defining distance which may better describe spatial spread. For example, diseases may transmit over a network, spreading first between well-connected populations through to poorly-connected populations. Populations that are geographically close to one another may not necessarily be well connected; distance in this model should instead be defined by the quantity of individuals moving between locations rather than their spatial proximity [6–12].

For human pathogens, transmission between distant populations has become increasingly common, as modern transportation now frequently allows individuals to move long distances over short periods of time [13, 14]. Recent work has repeatedly shown that incorporating human mobility into epidemic models allows for more accurate predictions of the rate and timing of disease invasion and spread [6, 15]. However, the impact of these various transportation networks on pathogen genetic structure is strongly dependent on spatial scale. Failure to detect similar patterns in structure across multiple spatial resolutions suggests that transmission processes are scale-dependent. For instance, although connectivity based on air travel volume between locations often correlates well with the trajectory of pathogen diffusion at the global scale [6], at finer resolutions, this mobility network may instead facilitate random mixing among hosts. These contrasting outcomes are influenced by attributes of the mobility network, which can include its size and span in relation to the geographic scale of interest, the number of hosts that utilize it and the regularity of host movements along it, as well as by the epidemiological properties of the pathogen.

Seasonal influenza A, a virus which causes major morbidity and mortality worldwide [16], provides an ideal system with which to compare the effects of human movement networks on pathogen population structure across various spatial scales. Although evidence suggests that the H3N2 subtype of influenza A (H3N2) is genetically structured as a source-sink metapopulation at the global scale [17, 18], it is generally accepted that no structure is present at finer spatial scales [19]. This is problematic for the design of containment strategies, since it suggests that the seasonal spread of influenza within countries is determined by stochastic processes and is therefore unpredictable. However, epidemiological reports and mortality statistics from influenza-like illness (ILI) data have revealed that spatial patterns do exist, with greater synchronization in epidemic peak timing observed between cities that are geographically close and exchange many commuters [20].

Studies tracking the intra-continental spread of influenza have thus far utilized ILI and excess mortality data, which cannot differentiate between the two subtypes of influenza A (H3N2 and H1N1) that circulate each season. Of the two viruses, H3N2 causes the most morbidity and mortality and has been dominant in six of the past ten influenza seasons in the United States (US) [21]. Its rapid evolution results in annual lineage replacement so that little genetic diversity is observed within seasons [22]. In contrast, lower substitution rates are common for seasonal H1N1, and seasons dominated by this subtype are generally characterized by reduced mortality and morbidity and increased genetic diversity among co-circulating lineages as compared to H3N2 [22–24]. It follows that these contrasting epidemiological dynamics could lead to subtype-specific population structure, but this hypothesis has not yet been formally tested.

We explored whether using alternative measures of distance can explain the population genetic structure of seasonal influenza A subtypes within the US. Since it has been shown that airline travel is important for the spread of influenza at the global scale [25] and that both commuter and airline travel contribute to the epidemiological dynamics of influenza within the US [20, 26], we investigated the roles that these transportation networks play at the regional scale. We constructed models of the US aviation and commuter networks and quantified interstate connectedness based on the daily number of individuals exchanged. If transmission is dominated by the local spread of influenza across the commuter network rather than long distance spread over the aviation network, we expect that sequences collected from pairs of states that are well-connected in terms of commuter flow will be more similar to each other than those collected from poorly-connected state pairs. To test this hypothesis, we obtained influenza sequences collected from 2003–2013 to compare associations of intra-seasonal pairwise genetic distances with geographic and network distance measures. Results indicate that population structure is indeed detectable, though this pattern is subtype specific.

## Results

### Transportation Networks

**Fig 1:**
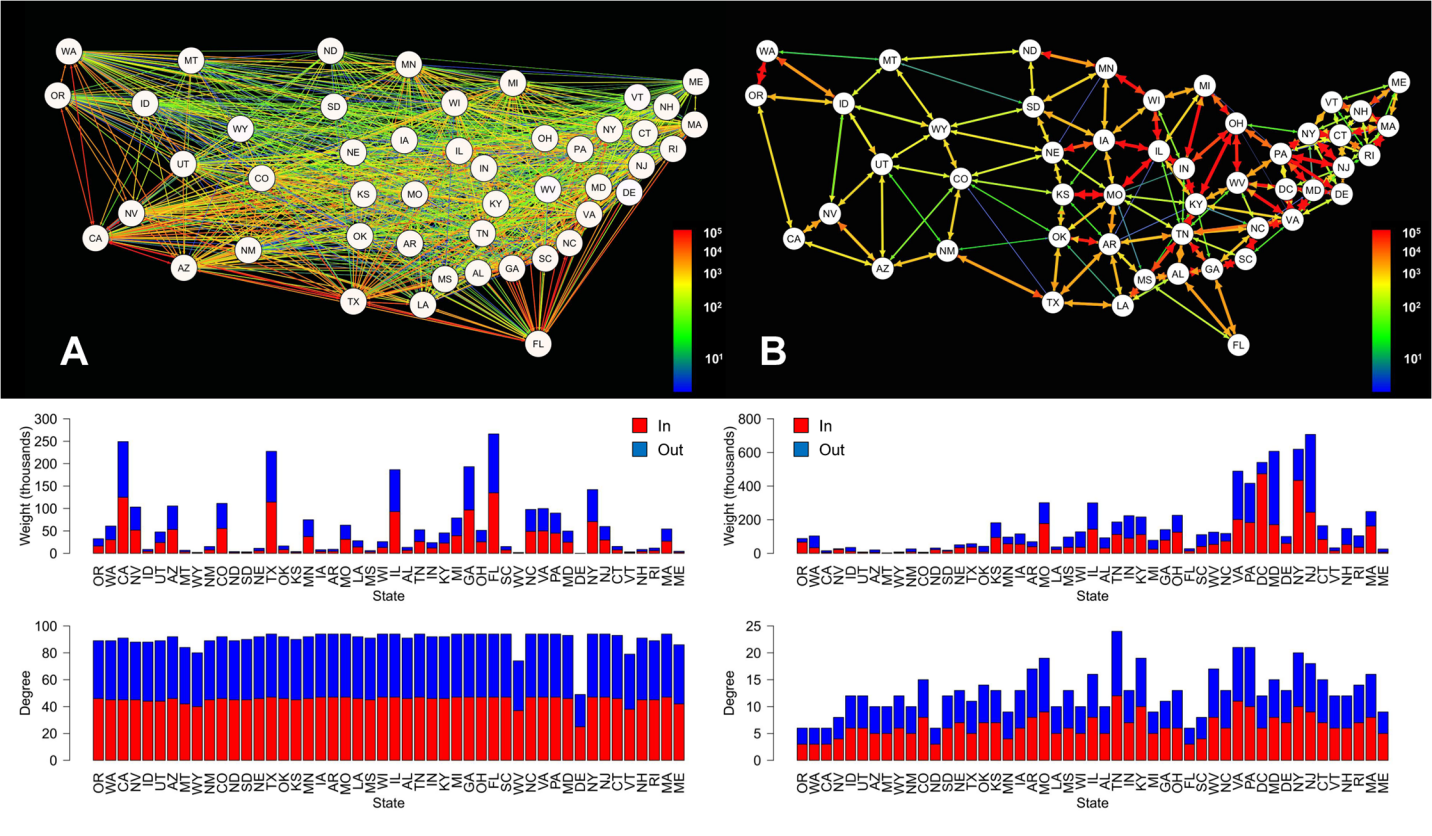
Aviation (A) and commuter (B) network models for the continental US. Edge colors represent the number of individuals traveling between each state pair per day. Bar plots directly below each network depict the weight (total number of individuals moving in (colored red) and out (colored blue) of a state; top) and degree (total number of connections in (colored red) and out (colored blue) of a state; bottom) for each of that network’s nodes, ordered from left to right by the longitude of each state’s population center.

Comparison of the aviation and commuting networks within the continental US revealed significant differences in their basic properties, despite the similarity in data resolution (travelers/day) (Fig 1). The aviation network, composed of 48 nodes connected by 2,160 edges, is highly homogeneous in terms of the total number of connections per node (degree) and has a high graph density (density = 0.96), reflecting that most states are directly connected to most other states. In contrast, connection weights differed greatly across state pairs. During the influenza season, approximately 1.6 million people travel along the interstate aviation network per day. In contrast, the commuter network is composed of 49 nodes and only 312 edges. Decreased graph density (density = 0.13) in comparison to the aviation network reflects that the commuter network is highly spatially organized, with connections generally only occurring between neighboring states. Over 3.8 million people travel daily across the interstate ground-travel commuter network, and interstate connections in the east tend to be stronger than those in the west.

**Fig 2:**
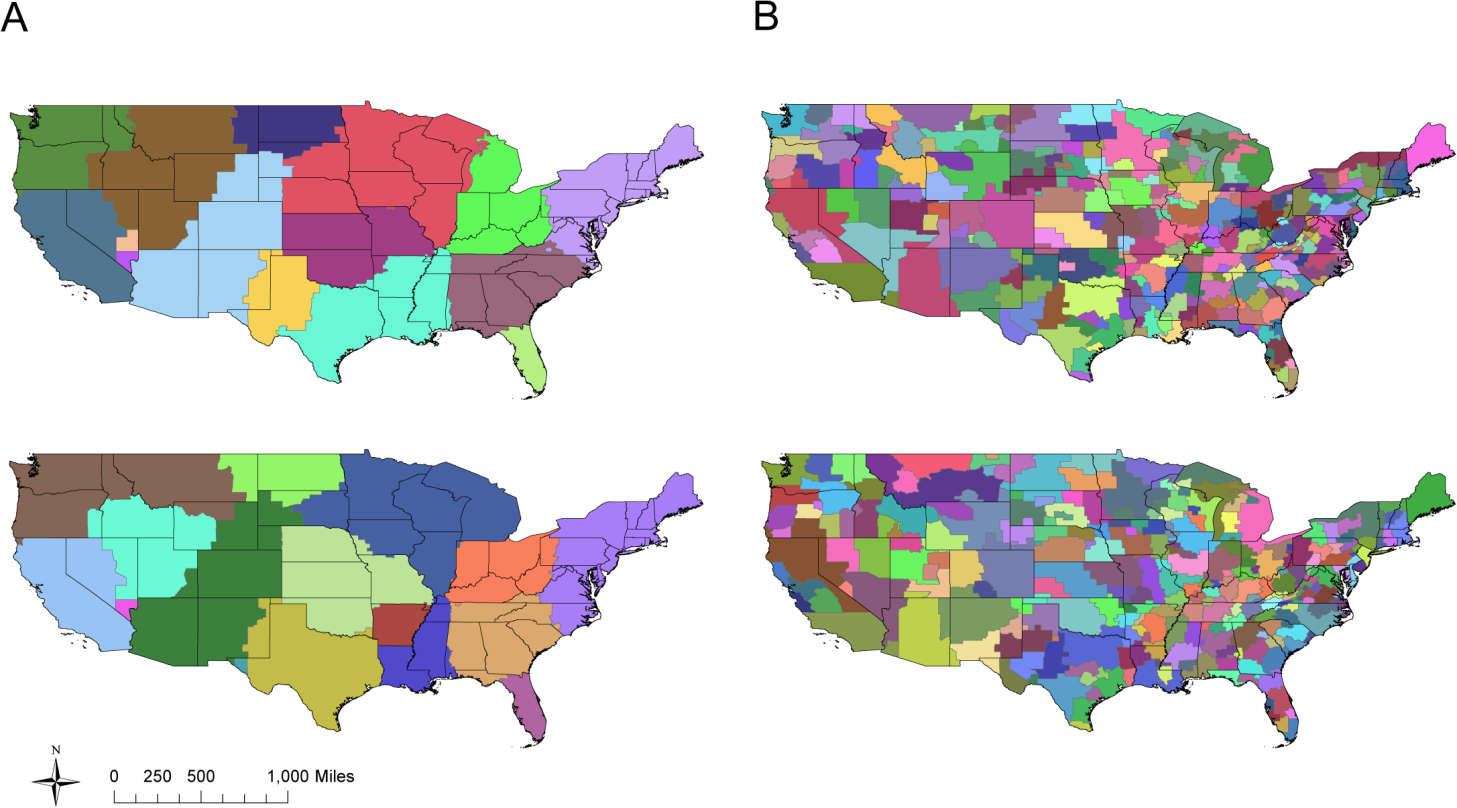
US commuting communities. Two realizations using the simulated annealing algorithm to partition the US into communities based on an unweighted network (A) and weighted network (B) of county-to-county commuter flows. Modularity is similar across all realizations for a given network type, although exact community compositions differ. In all realizations, community boundaries do not neatly coincide with state borders.

The community detection algorithm identified an average of 16 communities in the unweighted commuter network with an overall mean modularity of 0.55 (sd = 0.003) across the 1000 simulations (Fig 2A). In the weighted commuter network, an average of 135 communities were identified and mean modularity was 6.03 x 10^−4^ (sd = 1.33 x 10^−5^) (Fig 2B). For both networks, communities tend to span multiple states.

### Influenza A/H3N2

Phylogenetic trees were constructed for nine influenza seasons within the US from 2003–2004 to 2012–2013 (S1–S9 Figs); seven of these seasons contained clades for which we were able to evaluate population structure. The number of sequences available per season varied from147 in 2005–2006 to 1,276 in 2012–2013 and the number of states represented during a season varied from 29 in 2003–2004 to 49 in 2010–2011, 2011–2012, and 2012–2013 (S2 Table). The MRCA for each season existed from 1–3 years before present. Clades fitting the criteria for inclusion (see Materials & Methods) were not available from the 2004–2005 or 2008–2009 seasons. Detailed information on each season and clade tested obtained through the phylogenetic analysis can be found in S3 Table.

We detected a significant correlation between genetic distance and commuter distance for seven out of the 23 clades tested encompassing six out of seven seasons studied (Table 1). Mantel r correlation coefficients ranged from 0.09–0.38. We detected a significant correlation between genetic distance and geographic distance for five clades in four of the nine seasons (Mantel r: 0.14–0.33) and between genetic distance and aviation distance for two clades in two seasons (Mantel r: 0.31–0.42). Temporal distance between sequences, measured as the difference in number of days between collections, was never a significant predictor of population structure.

**Table 1:** Mantel r correlation coefficients measuring the association between matrices of genetic, temporal, geographic, aviation network and commuter network distance for H3N2 sequences.

	Correlation with genetic distance based on:			
Season	Temporal	Geographic	Aviation	Commuter
2003–2004				
*clade 1*	0.01 (p = 0.43)	0.20 (p = 0.03)	0.29 (p = 0.04)	***0*.*38 (p = 0*.*0009)***
*clade 2*	0.12 (p = 0.14)	0.07 (p = 0.16)	-0.13 (p = 0.89)	0.09 (p = 0.15)
2005–2006				
*clade 1*	0.02 (p = 0.39)	-0.17 (p = 0.98)	-0.01 (p = 0.53)	-0.04 (p = 0.67)
*clade 2*	0.00 (p = 0.48)	0.04 (p = 0.33)	*0*.*31 (p = 0*.02)*	*0*.*25 (p = 0*.02)*
2006–2007				
*clade 1*	-0.01 (p = 0.51)	*0*.*22 (p = 0*.009)*	-0.02 (p = 0.57)	***0*.*13 (p = 0*.*005)***
*clade 2*	0.11 (p = 0.06)	0.04 (p = 0.17)	0.01 (p = 0.45)	0.04 (p = 0.05)
2007–2008				
*clade 1*	-0.05 (p = 0.76)	-0.03 (p = 0.77)	0.12 (p = 0.07)	0.04 (p = 0.06)
*clade 2*	-0.15 (p = 0.88)	-0.08 (p = 0.74)	0.31 (p = 0.04)	-0.10 (p = 0.85)
*clade 3*	0.11 (p = 0.03)	-0.04 (p = 0.81)	0.06 (p = 0.21)	0.04 (p = 0.11)
*clade 4*	0.15 (p = 0.02)	*0*.*16 (p = 0*.0007)*	0.06 (p = 0.23)	***0*.*09 (p = 0*.*0001)***
*clade 5*	-0.02 (p = 0.70)	0.00 (p = 0.50)	0.00 (p = 0.49)	0.01 (p = 0.25)
2010–2011				
*clade 1*	-0.26 (p = 0.96)	-0.06 (p = 0.64)	-0.33 (p = 0.96)	-0.10 (p = 0.91)
*clade 2*	0.00 (p = 0.49)	-0.11 (p = 0.84)	-0.11 (p = 0.84)	-0.05 (p = 0.72)
2011–2012				
*clade 1*	-0.15 (p = 0.94)	***0*.*14 (p = 0*.*0002)***	0.27 (p = 0.03)	0.14 (p = 0.02)
*clade 2*	-0.07 (p = 0.80)	0.07 (p = 0.17)	0.08 (p = 0.23)	0.06 (p = 0.15)
*clade 3*	0.11 (p = 0.09)	***0*.*16 (p = 0*.*005)***	0.10 (p = 0.14)	*0*.*22 (p = 0*.0001)*
*clade 4*	0.19 (p = 0.13)	-0.15 (p = 0.84)	0.06 (p = 0.39)	0.03 (p = 0.40)
2012–2013				
*clade 1*	-0.06 (p = 0.65)	-0.13 (p = 0.76)	-0.10 (p = 0.69)	-0.15 (p = 0.91)
*clade 2*	-0.04 (p = 0.57)	0.13 (p = 0.28)	-0.16 (p = 0.77)	0.11 (p = 0.25)
*clade 3*	0.24 (p = 0.06)	-0.04 (p = 0.61)	-0.19 (p = 0.90)	0.05 (p = 0.23)
*clade 4*	0.01 (p = 0.44)	***0*.*33 (p = 0*.*005)***	*0*.*42 (p = 0*.003)*	***0*.*32 (p = 0*.*006)***
*clade 5*	0.03 (p = 0.33)	0.10 (p = 0.10)	-0.10 (p = 0.82)	***0*.*15 (p = 0*.*001)***
*clade 6*	-0.15 (p = 0.94)	0.07 (p = 0.09)	-0.07 (p = 0.70)	0.01 (p = 0.42)

Significant p-values are based on a Bonferroni correction, computed to account for multiple clade comparisons within a single season. When more than one distance metric is correlated with genetic distance, asterisks denote those metrics that remained significant after partial Mantel tests were conducted (at the p = 0.05 level).

For many clades, more than one distance measurement was significantly associated with genetic distance. After performing partial Mantel tests to account for these interactions, we found that commuter distance remained significant for four clades in four different seasons. Geographic distance remained significant for three clades in three different seasons and air travel remained significant for two clades in two different seasons.

### Influenza A/H1N1

Phylogenetic trees were constructed for six influenza seasons within the US from 2006–2007 to 2012–2013 (S10–S15 Figs); five of these seasons contained clades for which we were able to evaluate population structure. Correlations between genetic distance and commuter travel were detected for a greater proportion of clades when the analyses were repeated for H1N1 at the regional scale (Table 2). The number of sequences available per season varied from 165 in 2007–2008 to 371 in 2010–2011 and the number of states represented during a season varied from 16 in 2008–2009 to 48 in 2010–2011 (S2 Table). The MRCA for each season existed from 1–4 years before present (S4 Table). Detailed information on each season and clade tested obtained through the phylogenetic analysis can be found in S4 Table.

**Table 2:** Mantel r correlation coefficients measuring the association between matrices of genetic, temporal, geographic, aviation network and commuter network distance for H1N1 sequences.

	Correlation with genetic distance based on:			
Season	Temporal	Geographic	Aviation	Commuter
2006–2007				
*clade 1*	0.11 (p = 0.19)	*0*.*56 (p = 0*.0001)*	***0*.*32 (p = 0*.*005)***	***0*.*36 (p = 0*.*0003)***
*clade 2*	-0.09 (p = 0.76)	*0*.*44 (p = 0*.001)*	-0.21 (p = 0.98)	*0*.*38 (p = 0*.0001)*
*clade 3*	0.07 (p = 0.32)	0.13 (p = 0.12)	-0.17 (p = 0.86)	***0*.*20 (p = 0*.*002)***
2007–2008				
*clade 1*	-0.11 (p = 0.80)	0.07 (p = 0.23)	0.15 (p = 0.20)	***0*.*20 (p = 0*.*04)***
2010–2011				
*clade 1*	0.17 (p = 0.12)	0.28 (p = 0.03)	0.08 (p = 0.29)	***0*.*28 (p = 0*.*005)***
*clade 2*	-0.11 (p = 0.79)	-0.09 (p = 0.75)	-0.04 (p = 0.61)	0.03 (p = 0.30)
*clade 3*	0.00 (p = 0.49)	-0.16 (p = 0.99)	-0.12 (p = 0.92)	-0.04 (p = 0.83)
2011–2012				
*clade 1*	***0*.*31 (p = 0*.*0002)***	0.09 (p = 0.08)	0.04 (p = 0.35)	***0*.*17 (p = 0*.*007)***
*clade 2*	-0.08 (p = 0.93)	0.00 (p = 0.50)	0.01 (p = 0.46)	0.02 (p = 0.30)
2012–2013				
*clade 1*	-0.13 (p = 0.88)	0.04 (p = 0.37)	***0*.*26 (p = 0*.*04)*** ^***+***^	***0*.*22 (p = 0*.*03)*** ^***+***^

Significant p-values are based on a Bonferroni correction, computed to account for multiple clade comparisons within a single season. When more than one distance metric is correlated with genetic distance, asterisks denote those metrics that remained significant after partial Mantel tests were conducted (at the p = 0.05 level).

^+^ Neither metric remained significant after a partial mantel test was performed (at the p = 0.05 level).

Significant associations between genetic distance and commuter network distance occurred in all five seasons (Mantel r: 0.17–0.38). Both aviation network distance and geographic distance were associated with genetic distance in two clades in one and two different seasons, respectively (aviation Mantel r: 0.26–0.32; geographic Mantel r: 0.44–0.56) and temporal distance appeared significant in one clade from the 2011–2012 season (Mantel r: 0.31). After performing partial Mantel tests for clades in which more than one distance measure appeared significant, the commuter network remained significantly associated with genetic distance in five clades over four different seasons. In the 2012–2013 season, both commuter distance and aviation distance were significantly associated with genetic distance, although partial Mantel tests showed that neither remained significant when accounting for the other.

## Discussion

We have shown here the first evidence, to our knowledge, that population structure for seasonal influenza A is detectable at the scale of the continental US. Although all distance metrics were correlated with genetic distance for at least one clade, we found that the commuter network was more often associated with genetic distance than any other measure of spatial or network distance for the H1N1 subtype. Further, the association between genetic distance and the commuter network often remained significant after geographic distance was taken into account, demonstrating that the relative magnitude of host movement over space has a greater influence on the route of pathogen spread than the geographic proximity of sampling locations. In contrast, population structure was not detected in the majority of clades tested for H3N2, even though both geographic distance and commuter distance were, at times, correlated with genetic distance. This discrepancy suggests that epidemiological differences between H3N2 and H1N1 affect our ability to detect population structure of influenza within a season at this spatial scale.

Striking differences in the epidemiological dynamics of seasons dominated by H3N2 and H1N1 have been previously documented [20]. The rapid bicoastal spread of H3N2 should obscure our ability to detect patterns based on geography or commuting if long distance transmission (through the aviation network, for example) quickly moves the virus between spatially distant localities. Models of the effect of R_o_ on the spread of influenza across the US and its implications for spatial synchrony have previously shown that ILI cases in cities across the entire US tend to peak around the same time when influenza spread is rapid [20]. In contrast, seasons dominated by H1N1 tend to be milder and characterized by slower dispersal. The slower nationwide spread of H1N1 may facilitate the detection of population structure if H1N1 is allowed to diffuse over short-range connections once it is introduced into a new geographic area. Differences in the rate of spread between multiple clades from the same season could possibly account for our failure to consistently detect these patterns across all lineages. The degree of matching between vaccine strains and circulating lineages could also potentially act to reduce transmission so that the commuter network would be able to exert a sufficiently strong influence in structuring the influenza population. However, there are multiple other factors that vary seasonally which could confound this relationship including, for example, vaccine efficacy, availability, population coverage, or age structure of vaccinated individuals. Models combining genetic and epidemiological data may be able to shed light on this proposed relationship but have only recently been utilized [22, 27–29]; adding a spatially explicit component to these models remains an area for future research [30].

An investigation into the two circulating lineages of influenza B, a virus which causes milder disease than either subtype of influenza A [31], would provide an interesting point of comparison to our findings. As population structure based on commuter travel is more pronounced for A/H1N1, we might expect it to also be evident for influenza B. However, as influenza B primarily affects children [31], the role of commuters in transmission may be reduced such that structure is instead based on geographic distance. Interestingly, recent work on the epidemiology of influenza B in China showed that the Yamagata lineage tends to infect older age groups than the Victoria lineage [30]; examining these lineages separately may reveal differences in population structure patterns and/or modes of spread within the US. So far, little research to date has been done on the spreading patterns of influenza B and unfortunately, few sequences are publicly available on GenBank, as compared to either influenza A subtype.

Apart from biological explanations, uneven sampling may also be responsible for our inability to detect population structure in more seasons, or across all clades within a season. Differences in the number of sequences available for each season are a product of inconsistent sampling among states within a season and differential severity of the influenza virus across seasons. For example, the number of testing facilities differs by state and the quantity of samples sequenced has historically been a function of individual laboratory capacity [32]. Additionally, seasons that are characterized by more severe influenza subtypes or poor vaccine performance tend to yield more sequences [33]. Furthermore, seasons dominated by H3N2 generally result in higher rates of morbidity and mortality than those dominated by H1N1 or influenza B [34]. Better virologic surveillance in less populous locations that are not travel hubs (i.e. in states outside of New York or Texas for example, which often contributed an excess of sequences per season) would enable us to better catalog influenza diversity outside of major cities and potentially increase our power to detect spatial patterns in this genetic data.

The correlations we detected are not as strong as those observed between these same distance metrics and epidemiological data [20]. First, we caution against the interpretation of the Mantel r value as a standard correlation coefficient such as that calculated from a linear regression. Mantel r correlation coefficients are typically much lower than those reported for other statistical tests, owing to the comparison of distances between variables rather than their absolute values. Further, due to differences in the calculation of the sum of squares statistic, a standard R^2^ cannot be derived from this value for use as a measure of the variation in the dependent variable explained by the predictor variable [35]. However, the discrepancy in correlation strength may be due to differences in the underlying processes producing these associations. For example, epidemics in different locations could follow similar trajectories in terms of peak timing if one directly seeded the other; however, this could also result if the epidemics were initiated at similar times due to similarities between states in population size or climate. In contrast, correlations between locations based on genetic distance should only arise if epidemics in one location were directly seeded by the other. In systems such as this, where long distance dispersal is prevalent, noise due to the circulation of multiple lineages in a single location likely obscures fine scale signatures of diffusion [19]. We have attempted to account for this noise by using phylogenetic methods to aggregate samples by clade so that only sequences derived from the same introduction, and therefore the same genetic lineage, are compared. However, uncertainty surrounding divergence dates always exists; that we are able to detect any correlation at all is surprising, as none have been found previously [19].

At this spatial scale, the ability of the commuter network to exert a structuring influence on regional influenza populations is directly counteracted by the aviation network, which instead acts to create a randomly mixed viral population. These opposing effects stem from differences in the predictability of transmission processes within the two transportation networks. The commuter network is highly spatially organized, with 99% of commutes occurring over distances less than 150 miles (242 km). Individuals travel along the commuter network on a daily basis, increasing both the probability of transmission to coworkers and any others with whom an infected individual encounters regularly. These movements along the network lead to a genotypic cline; viral sequences collected from nodes separated by less traveled paths appear less similar than those collected from node pairs that are well connected. In contrast, movement along the aviation network is less predictable. Although individuals traveling by air are likely to remain at their destination for several days, these trips are not likely to reoccur multiple times within a season, thus counteracting the structuring effects of routine commuting. That we find any structure at all is an indication that daily travel to and from work is an important route of interstate spread for seasonal influenza. Although infection pathways can be linked to air travel at the global scale [25], at the regional scale, air transportation likely functions to move the virus long distances into new areas that have not yet been invaded [15] where it then undergoes short-range dispersal by commuters.

In our characterization of the US commuting network, we were able to partition the US into communities of high modularity based on county-to-county connections. While partitioning these communities using daily total commuter flow estimates (weighted networks) resulted in weakly supported subdivisions that provided little information about human mobility, analyzing county-to-county connections based on the presence or absence of commuter movements (unweighted networks) resulted in subdivisions of high modularity. These communities tended to span multiple states, lending further support to the hypothesis that interstate commuter travel is a viable means of influenza transmission. More importantly, states tended to be part of multiple communities, suggesting that aggregation of sequences by state may be somewhat arbitrary and that finer scale location data for sequences is needed. Our results are in good agreement with previous characterizations of US community structure [36], which have used currency movement as a proxy for human mobility. Since human movement tends to be limited to spatially compact groups of counties and repeated studies have shown that commuters are responsible for a significant portion of transmission, grouping sequences by commuting community rather than by state may provide a more accurate method of determining which sequences are most likely to be closely related [25]; comparing these sequences sets with network distance may then yield stronger and more consistent relationships between genetic distance and the commuter network. Further, these communities may in fact provide a measure of the spatial extent over which commuting is responsible for the majority of transmission, with air travel operating to transfer influenza lineages between communities. Unfortunately, the spatial data associated with most publicly available sequences is currently limited to the US state of collection. Since commuting communities are defined by county-level associations, the availability of only state-level reporting hinders our ability to analyze the data at this alternative resolution. Clearly, there is a need for more informative spatial data to be made publicly available in order to facilitate analyses using more natural geographic groupings, rather than those arbitrarily imposed by political boundaries.

The results from our study complement recent findings that the aviation network plays an important role in the world-wide transmission of seasonal influenza. While the aviation network is undoubtedly of importance in structuring populations at the global scale, we find that, when population structure is detectable, it is the commuter network that is of greater importance at more regional scales. Host movement governs disease transmission patterns, and distinct modes of movement by discrete segments of the population can have varying levels of importance. While the magnitude of the correlations we detected was not overly strong, this may not be the case at finer geographic resolutions, such as within commuting communities or at the state-wide level, or at finer temporal resolutions, such as during the onset of an epidemic before any appreciable long distance transmission has occurred. While commuters living near state borders likely accounted for much of the interstate connectivity measured by our metric, at the intrastate scale, commuters moving between counties may comprise a larger segment of the population. However, local movement networks, such as that of children being transported to and from school, may prove more important in structuring influenza populations at this scale. Previous work has suggested that children are responsible for much of the transmission within communities [37]. Future work is needed to further elucidate the scales at which different movement patterns contribute most to disease transmission.

## Materials and Methods

### Sequence Data

In total, 3,063 influenza A/H3N2, and 1,366 A/H1N1hemagglutinin sequences collected from 2003–2013 in the continental US were obtained from the National Center for Biotechnology Information Influenza Virus Resource for use in this analysis [38]. Collection date was used to assign each sequence to a season, with seasons defined as occurring from Oct 1 to May 31. We restricted our analyses to seasons containing more than 90 sequences that were collected in at least 10 different states. This criterion was based on a natural break in the data, as seasons that did not fit this criterion tended to have fewer than 30 sequences that were restricted in their geographic distribution. This criterion was therefore necessary to achieve representative seasonal datasets in terms of sequence diversity and geographic coverage. For example, only 11 H3N2 sequences were available from the 2009–2010 season since the H1N1 subtype was dominant; this season was therefore excluded from all analyses of H3N2 data. Using this criterion, we were able to evaluate influenza phylogenetic structure in nine seasons for H3N2 (2003–2004 to 2012–2013, excluding 2009–2010), and six seasons for H1N1 (2006–2007 to 2012–2013, excluding the 2009–2010 pandemic; see below). For each subtype, isolates came from all locations within the 48 continental states and the District of Columbia. The specific set of states represented varied seasonally and with each subtype. GenBank accession numbers for all sequences used in this study, as well their location and collection dates are listed in S1 Table.

### Phylogenetic Analysis

Sequences were aligned using MUSCLE in Geneious [39] and the HA1 domain was extracted for use in all analyses (H3N2: 987 nt, H1N1: 1701 nt). Seasonal influenza is introduced into the US multiple times over the course of the season [19]. To account for these multiple introductions, phylogenetic trees were inferred separately for each season using a bayesian framework in the program BEAST [40, 41]. To construct phylogenies, we used the SRD06 codon position model to accommodate different substitution rates for the first and second versus the third codon position, with the HKY85 substitution model applied over these two codon positions [42]. For two seasons for which an extremely large number of sequences were available, H3N2 2007–2008 and H3N2 2012–2013, we down-sampled from states that contributed exceptionally large numbers of sequences. For the H3N2 2007–2008 season, the GTR+I+G model used, as convergence could not be achieved using the codon position model. Trees were constructed using a strict molecular clock, with an exponential growth tree prior and relatively uninformative priors on all phylogenetic parameters except for the substitution rate, for which we used a lognormal prior with mean = 0.0055 (sd = 0.7) substitutions/site/year for H3N2 sequences [43] and mean = 0.0018 substitutions/site/year (sd = 0.4) for H1N1 sequences [22]. MCMC chains were run until convergence was reached and a maximum clade credibility tree was annotated after removing the first 10% of the sampled trees as a burn-in. We defined clades as groups of at least 20 sequences stemming from a node with a posterior probability of > 0.9. We corrected for independent introductions into the US by choosing clades for which the entire HPD interval for the divergence time of the MRCA did not fall more than three months before the beginning of the flu season. This time limit was chosen as it was generally the most recent time period for which high posterior support could be obtained for clades. Since several clades fitting these criteria were often identified within a single season, we used a Bonferroni correction within seasons, based on the number of clades identified for a season to account for these multiple comparisons.

For each clade analyzed, pairwise genetic distances were calculated as the proportion of sites that differed between each pair of sequences. To ensure that the choice of genetic distance metric did not affect our results, analyses were repeated using the evolutionary substitution models available in the R package APE [44]. The results remained the same regardless of the distance metric chosen, so we chose to present those results obtained using the raw pairwise distance measure. Pairwise spatial distances were calculated based on the great circle distance between state population centers.

The 2008–2009 and 2009–2010 seasons presented a special case for H1N1, as a new pandemic lineage emerged in the spring of 2009 that differed markedly from the currently and previously circulating H1N1 lineages. As epidemiological dynamics of influenza pandemics differ substantially from those of annual seasonal epidemics [24], sequences from the pandemic lineage in the 2008–2009 season, as well as the entire 2009–2010 season, were excluded from all analyses. To distinguish between antigenically distinct pandemic isolates and the previously circulating H1N1 viruses, a phylogenetic tree was inferred for the 2008–2009 season using a neighbor-joining algorithm. Two clades were immediately obvious, each encompassing distinct time periods during the influenza season that corresponded well with the circulation times of the epidemic and pandemic lineages. Using the A/California/07/2009 strain of pandemic H1N1 (GenBank accession: FJ981613) as a reference, sequences were classified and excluded accordingly.

### Transportation Network Models

Data on the origin, destination and passenger volume of airline routes within the continental US during October to March from 2003–2012 were obtained from the Office of Airline Information, Bureau of Transportation Statistics, Research and Innovative Technology Administration [45]. Data were restricted to this time period to best represent human movement during the US influenza season, which occurs during the fall and winter and generally peaks anytime from late November to March [46]. Passenger movement data for each airport were aggregated by state, so that each state was considered a node in each season-specific aviation network model. Data on intra-state passenger movement was excluded. Each seasonal aviation network model therefore contained 48 nodes (all continental US states), with directed edges weighted by the number of daily passengers traveling between each unique state pair during the influenza season. Because there are no airports located within the District of Columbia, sequences from this location were excluded for the aviation analysis. To ensure that this did not affect our results, we repeated the analysis with sequences from the District of Columbia coded as being from Maryland or from Virginia; no qualitative differences in the Mantel test results were observed. To facilitate summary comparisons with the commuter network model, a single aviation network model was also constructed based on the average number of passengers exchanged per day between states over all ten winter seasons

Data on the origin, destination and commuter volume between all US county pairs collected during the 2000 census were available from the US Census Bureau [47]. Commuter volume estimates were based on census participant responses when questioned on the county location worked in most often during the preceding week. As commuting data are intended as a proxy for long-distance influenza transmission occurring by means other than airline travel, commutes exceeding 150 miles (242 km) were excluded from the final commuter network (and accounted for only 0.07% of county-to-county movements). To assess the sensitivity of our results to this assumption, the analyses were repeated using the full commuter network, which included journeys of all distances. For all but one H3N2 clade, and two H1N1 clades tested, results were similar regardless of whether the full or reduced commuter network was used; we therefore only present the results using the reduced commuter matrix. Intra-state commutes were also excluded. Data on commuter movements between counties were aggregated by state so that the final commuter network model contained 49 nodes (all continental US states and the District of Columbia) with directed edges weighted by the number of daily commuters traveling between each unique state pair.

For each transportation network model, each node corresponds to a single state, and each edge represents the total daily number of either commuter or air travel passengers moving between those states. To compare the basic properties of the two different transportation networks, node degrees and graph density metrics were calculated. Node degree is defined as the total number of connections per node and graph density is calculated as the proportion of edges present in the graph out of the maximum number of edges possible.

To assess the validity of aggregating sequences by state, a community detection algorithm based on simulated annealing [48–50] was run for both unweighted and weighted networks of county level commuter movements. We used the methods described by Thiemann and colleagues [36] to compute 1000 partitions of high modularity to determine the underlying community structure for each network. Communities in this context refer to groups of nodes which have stronger ties internally than externally. The community structure of a network can be summarized by network modularity, Q, which measures the overall magnitude of difference between partitions [49]. The modularity value of a particular set of partitions is calculated by taking the difference between the fraction of total connections occurring within communities and the expected value of the fraction of total edges occurring within communities in a network of identical community partitions with randomized connections between nodes. Q is bounded between 0–1, with Q = 0 indicating that that the community subdivisions provide no more information than that of a random partitioning of nodes.

Associations between pairwise genetic distances and measures of geographic and network distance were assessed individually for each season through the use of Mantel’s test [51]. In order to conduct these tests, connection weights between states for each of the transportation networks were symmetrized by taking the sum of both connecting edges. Mantel tests were performed on both the raw connectivity distance matrices (constructed using the raw number of people traveling between states) and connectivity distance matrices constructed using the effective distance metric developed by Brockman et al. [6]. This metric is based on the proportion of individuals commuting between states in relation to the total number of commuters in the entire US. Results were similar regardless of the connectivity metric chosen; all results presented are those results obtained using raw connectivity. To account for multiple comparisons, a Bonferroni correction was applied to the results when multiple clades were tested from a single season. When multiple distance metrics (geographic, aviation or commuter distances) were significantly correlated with genetic distance for a single clade, partial Mantel tests were performed to account for these interactions. Partial Mantel tests allow for the comparison of two matrices while controlling for the effects of a third by regressing the two matrices of interest on the third matrix, and performing a standard Mantel tests using these residuals. Results of the partial Mantel tests were used to identify the distance metric responsible for driving patterns of population structure.

## Supporting Information

S1 Table Accession numbers, locations and collection dates of all sequences used in this study. (CSV)

S2 Table Number of sequences per season and number of locations (US states) represented for influenza A/H3N2 and A/H1N1 by season. Numbers in parentheses indicate the total number of publicly available sequences for those seasons; because of the extremely large sample size as compared to other seasons, subsamples were taken from states in these seasons that contributed an excessive number of sequences.
(DOCX)

S3 Table Summary of epidemiological and evolutionary dynamics of H3N2 epidemics based on phylogenetic analyses of each influenza season. ‘Root Height’ is measured in years before present, with the present time equal to the latest sampling date. ‘Clock rate’ is measured in substitutions/site/year. ‘Sequences’ represents the number of sequences analyzed per clade and ‘Locations’ represents the number of states these sequences were collected from.
(DOCX)

S4 Table Summary of epidemiological and evolutionary dynamics of H1N1 epidemics based on phylogenetic analyses of each influenza season. ‘Root Height’ is measured in years before present, with the present time equal to the latest sampling date. ‘Clock rate’ is measured in substitutions/site/year. ‘Sequences’ represents the number of sequences analyzed per clade and ‘Locations’ represents the number of states these sequences were collected from.
(DOCX)

S1 Fig Phylogenetic tree estimated using influenza A/H3N2 HA sequences sampled from a single subtype within the 2003–2004 influenza season in the US using a Bayesian method. Clades used for association tests are highlighted in green. Posterior probability values (>0.9) are labeled for nodes leading to clades used in the correlation analysis. Horizontal axis is measured in years.
(PDF)

S2 Fig Phylogenetic tree estimated using influenza A/H3N2 HA sequences sampled from a single subtype within the 2004–2005 influenza season in the US using a Bayesian method. Horizontal axis is measured in years.
(PDF)

S3 Fig Phylogenetic tree estimated using influenza A/H3N2 HA sequences sampled from a single subtype within the 2005–2006 influenza season in the US using a Bayesian method. Clades used for association tests are highlighted in green. Posterior probability values (>0.9) are labeled for nodes leading to clades used in the correlation analysis. Horizontal axis is measured in years.
(PDF)

S4 Fig Phylogenetic tree estimated using influenza A/H3N2 HA sequences sampled from a single subtype within the 2006–2007 influenza season in the US using a Bayesian method. Clades used for association tests are highlighted in green. Posterior probability values (>0.9) are labeled for nodes leading to clades used in the correlation analysis. Horizontal axis is measured in years.
(PDF)

S5 Fig Phylogenetic tree estimated using influenza A/H3N2 HA sequences sampled from a single subtype within the 2007–2008 influenza season in the US using a Bayesian method. Clades used for association tests are highlighted in green. Posterior probability values (>0.9) are labeled for nodes leading to clades used in the correlation analysis. Horizontal axis is measured in years.
(PDF)

S6 Fig Phylogenetic tree estimated using influenza A/H3N2 HA sequences sampled from a single subtype within the 2008–2009 influenza season in the US using a Bayesian method. Horizontal axis is measured in years.
(PDF)

S7 Fig Phylogenetic tree estimated using influenza A/H3N2 HA sequences sampled from a single subtype within the 2010–2011 influenza season in the US using a Bayesian method. Clades used for association tests are highlighted in green. Posterior probability values (>0.9) are labeled for nodes leading to clades used in the correlation analysis. Horizontal axis is measured in years.
(PDF)

S8 Fig Phylogenetic tree estimated using influenza A/H3N2 HA sequences sampled from a single subtype within the 2011–2012 influenza season in the US using a Bayesian method. Clades used for association tests are highlighted in green. Posterior probability values (>0.9) are labeled for nodes leading to clades used in the correlation analysis. Horizontal axis is measured in years.
(PDF)

S9 Fig Phylogenetic tree estimated using influenza A/H3N2 HA sequences sampled from a single subtype within the 2012–2013 influenza season in the US using a Bayesian method. Clades used for association tests are highlighted in green. Posterior probability values (>0.9) are labeled for nodes leading to clades used in the correlation analysis. Horizontal axis is measured in years.
(PDF)

S10 Fig Phylogenetic tree estimated using influenza A/H1N1 HA sequences sampled from a single subtype within the 2006–2007 influenza season in the US using a Bayesian method. Clades used for association tests are highlighted in green. Posterior probability values (>0.9) are labeled for nodes leading to clades used in the correlation analysis. Horizontal axis is measured in years.
(PDF)

S11 Fig Phylogenetic tree estimated using influenza A/H1N1 HA sequences sampled from a single subtype within the 2007–2008 influenza season in the US using a Bayesian method. Clades used for association tests are highlighted in green. Posterior probability values (>0.9) are labeled for nodes leading to clades used in the correlation analysis. Horizontal axis is measured in years.
(PDF)

S12 Fig Phylogenetic tree estimated using influenza A/H1N1 HA sequences sampled from a single subtype within the 2008–2009 influenza season in the US using a Bayesian method. Horizontal axis is measured in years.
(PDF)

S13 Fig Phylogenetic tree estimated using influenza A/H1N1 HA sequences sampled from a single subtype within the 2010–2011 influenza season in the US using a Bayesian method. Clades used for association tests are highlighted in green. Posterior probability values (>0.9) are labeled for nodes leading to clades used in the correlation analysis. Horizontal axis is measured in years.
(PDF)

S14 Fig Phylogenetic tree estimated using influenza A/H1N1 HA sequences sampled from a single subtype within the 2011–2012 influenza season in the US using a Bayesian method. Clades used for association tests are highlighted in green. Posterior probability values (>0.9) are labeled for nodes leading to clades used in the correlation analysis. Horizontal axis is measured in years.
(PDF)

S15 Fig Phylogenetic tree estimated using influenza A/H1N1 HA sequences sampled from a single subtype within the 2012–2013 influenza season in the US using a Bayesian method. Clades used for association tests are highlighted in green. Posterior probability values (>0.9) are labeled for nodes leading to clades used in the correlation analysis. Horizontal axis is measured in years.
(PDF)

## References

[1] Biek R, Henderson JC, Waller LA, Rupprecht CE, Real LA. A high-resolution genetic signature of demographic and spatial expansion in epizootic rabies virus. Proc Natl Acad Sci U S A. 2007;104(19):7993–8. 17470818 10.1073/pnas.0700741104 PMC1876560

[2] Real LA, Henderson JC, Biek R, Snaman J, Jack TL, Childs JE, et al Unifying the spatial population dynamics and molecular evolution of epidemic rabies virus. P Natl Acad Sci USA. 2005;102(34):12107–11. 16103358 10.1073/pnas.0500057102 PMC1186024

[3] Walsh PD, Biek R, Real LA. Wave-like spread of Ebola Zaire. Plos Biol. 2005;3(11):1946–53. 10.1371/journal.pbio.0030371 PMC1262627 16231972

[4] Epperson BK. Geographical Genetics. Princeton: Princeton University Press; 2003.

[5] Wright S. Isolation by distance. Genetics. 1943;28(2):114–38. 17247074 10.1093/genetics/28.2.114 PMC1209196

[6] Brockmann D, Helbing D. The hidden geometry of complex, network-driven contagion phenomena. Science. 2013;342(6164):1337–42. 10.1126/science.1245200 24337289

[7] Rvachev LA, Longini IM. A mathematical model for the global spread of influenza. Math Biosci. 1985;75(1):3–22.

[8] Hufnagel L, Brockmann D, Geisel T. Forecast and control of epidemics in a globalized world. P Natl Acad Sci USA. 2004;101(42):15124–9. 15477600 10.1073/pnas.0308344101 PMC524041

[9] Colizza V, Barrat A, Barthelemy M, Vespignani A. The modeling of global epidemics: stochastic dynamics and predictability. Bulletin of mathematical biology. 2006;68(8):1893–921. 17086489 10.1007/s11538-006-9077-9 PMC7089095

[10] Colizza V, Barrat A, Barthelemy M, Vespignani A. The role of the airline transportation network in the prediction and predictability of global epidemics. P Natl Acad Sci USA. 2006;103(7):2015–20. 16461461 10.1073/pnas.0510525103 PMC1413717

[11] Tatem AJ, Rogers DJ, Hay SI. Global transport networks and infectious disease spread In: Simon I. Hay AG, David JR, editors. Advances in Parasitology: Academic Press; 2006 p. 293–343. 10.1016/S0065-308X(05)62009-X PMC3145127 16647974

[12] Gomes MFC, Pastore y Piontti A, Rossi L, Chao D, Longini I, Halloran ME, et al Assessing the International Spreading Risk Associated with the 2014 West African Ebola Outbreak. PLoS Currents. 2014;6:ecurrents.outbreaks.cd818f63d40e24aef769dda7df9e0da5. 10.1371/currents.outbreaks.cd818f63d40e24aef769dda7df9e0da5 PMC4169359 25642360

[13] Jones KE, Patel NG, Levy MA, Storeygard A, Balk D, Gittleman JL, et al Global trends in emerging infectious diseases. Nature. 2008;451(7181):990–3. 10.1038/nature06536 18288193 PMC5960580

[14] Smith KF, Guegan JF. Changing Geographic Distributions of Human Pathogens. Annu Rev Ecol Evol S. 2010;41:231–50.

[15] Balcan D, Colizza V, Goncalves B, Hu H, Ramasco JJ, Vespignani A. Multiscale mobility networks and the spatial spreading of infectious diseases. P Natl Acad Sci USA. 2009;106(51):21484–9. 10.1073/pnas.0906910106 20018697 PMC2793313

[16] Simonsen L. The global impact of influenza on morbidity and mortality. Vaccine. 1999;17:S3–S10. 10471173 10.1016/s0264-410x(99)00099-7

[17] Russell CA, Jones TC, Barr IG, Cox NJ, Garten RJ, Gregory V, et al The global circulation of seasonal influenza A (H3N2) viruses. Science. 2008;320(5874):340–6. 10.1126/science.1154137 18420927

[18] Bahl J, Nelson MI, Chan KH, Chen RB, Vijaykrishna D, Halpin RA, et al Temporally structured metapopulation dynamics and persistence of influenza A H3N2 virus in humans. P Natl Acad Sci USA. 2011;108(48):19359–64. 10.1073/pnas.1109314108 22084096 PMC3228450

[19] Nelson MI, Edelman L, Spiro DJ, Boyne AR, Bera J, Halpin R, et al Molecular epidemiology of A/H3N2 and A/H1N1 influenza virus during a single epidemic season in the United States. Plos Pathog. 2008;4(8). 10.1371/journal.ppat.1000133 PMC2495036 18725925

[20] Viboud C, Bjornstad ON, Smith DL, Simonsen L, Miller MA, Grenfell BT. Synchrony, waves, and spatial hierarchies in the spread of influenza. Science. 2006;312(5772):447–51. 16574822 10.1126/science.1125237

[21] Centers for Disease Control and Prevention. FluView: A Weekly Influenza Surveillance Report Prepared by the Influenza Division. Atlanta [Accessed 2014 May]; http://www.cdc.gov/flu/weekly/pastreports.htm.

[22] Ferguson NM, Galvani AP, Bush RM. Ecological and immunological determinants of influenza evolution. Nature. 2003;422(6930):428–33. 12660783 10.1038/nature01509

[23] Rambaut A, Pybus OG, Nelson MI, Viboud C, Taubenberger JK, Holmes EC. The genomic and epidemiological dynamics of human influenza A virus. Nature. 2008;453(7195):615–9. 10.1038/nature06945 18418375 PMC2441973

[24] Simonsen L, Viboud C, Taylor R, Miller M. The Epidemiology of Influenza and Its Control In: Rappuoli R, Del Giudice G, editors. Influenza Vaccines for the Future: Springer Basel; 2011 p. 27–54.

[25] Lemey P, Rambaut A, Bedford T, Faria N, Bielejec F, Baele G, et al Unifying Viral Genetics and Human Transportation Data to Predict the Global Transmission Dynamics of Human Influenza H3N2. Plos Pathog. 2014;10(2):e1003932 10.1371/journal.ppat.1003932 24586153 PMC3930559

[26] Brownstein JS, Wolfe CJ, Mandl KD. Empirical evidence for the effect of airline travel on inter-regional influenza spread in the United States. PLoS medicine. 2006;3(10):e401 16968115 10.1371/journal.pmed.0030401 PMC1564183

[27] Ratmann O, Donker G, Meijer A, Fraser C, Koelle K. Phylodynamic inference and model assessment with approximate bayesian computation: influenza as a case study. Plos Comput Biol. 2012;8(12). 10.1371/journal.pcbi.1002835 PMC3531293 23300420

[28] Koelle K, Cobey S, Grenfell B, Pascual M. Epochal evolution shapes the phylodynamics of interpandemic influenza A (H3N2) in humans. Science. 2006;314(5807):1898–903. 17185596 10.1126/science.1132745

[29] Koelle K, Khatri P, Kamradt M, Kepler TB. A two-tiered model for simulating the ecological and evolutionary dynamics of rapidly evolving viruses, with an application to influenza. J R Soc Interface. 2010;7(50):1257–74. 10.1098/rsif.2010.0007 20335193 PMC2894885

[30] Bedford T, Rambaut A, Pascual M. Canalization of the evolutionary trajectory of the human influenza virus. Bmc Biol. 2012;10 10.1186/1741-7007-10-10 22546494 PMC3373370

[31] Atkinson W, Wolfe S, Hamborsky J, editors. Epidemiology and Prevention of Vaccine-Preventable Diseases. 12 ed. Washington DC: Public Health Foundation; 2012.

[32] Association of Public Health Laboratories, Centers for Disease Control and Prevention. Influenza Virologic Surveillance Right Size Roadmap. 2013. http://www.aphl.org/aphlprograms/infectious/influenza/Pages/Influenza-Virologic-Surveillance-Right-Size-Roadmap.aspx

[33] Centers for Disease Control and Prevention. FluView Web Portal: National and Regional Level Outpatient Illness and Viral Surveillance. [Accessed 2014 Feb]; http://gis.cdc.gov/grasp/fluview/fluportaldashboard.html.

[34] Simonsen L, Reichert TA, Viboud C, Blackwelder WC, Taylor RJ, Miller MA. Impact of influenza vaccination on seasonal mortality in the US elderly population. Arch Intern Med. 2005;165(3):265–72. 15710788 10.1001/archinte.165.3.265

[35] Legendre P, Fortin MJ. Comparison of the Mantel test and alternative approaches for detecting complex multivariate relationships in the spatial analysis of genetic data. Molecular ecology resources. 2010;10(5):831–44. 10.1111/j.1755-0998.2010.02866.x 21565094

[36] Thiemann C, Theis F, Grady D, Brune R, Brockmann D. The structure of borders in a small world. PloS one. 2010;5(11):e15422 10.1371/journal.pone.0015422 21124970 PMC2987795

[37] Medlock J, Galvani AP. Optimizing influenza vaccine distribution. Science. 2009;325(5948):1705–8. 10.1126/science.1175570 19696313

[38] Bao Y, Bolotov P, Dernovoy D, Kiryutin B, Zaslavsky L, Tatusova T, et al The Influenza Virus Resource at the National Center for Biotechnology Information. Journal of Virology. 2008;82(2):596–601. 17942553 10.1128/JVI.02005-07 PMC2224563

[39] Biomatters. Geneious version 5.6.2.

[40] Drummond AJ, Ho SYW, Phillips MJ, Rambaut A. Relaxed phylogenetics and dating with confidence. Plos Biology. 2006;4(5):699–710. 10.1371/journal.pbio.0040088 PMC1395354 16683862

[41] Drummond AJ, Rambaut A. BEAST: Bayesian evolutionary analysis by sampling trees. Bmc Evol Biol. 2007;7:214 17996036 10.1186/1471-2148-7-214 PMC2247476

[42] Shapiro B, Rambaut A, Drummond AJ. Choosing appropriate substitution models for the phylogenetic analysis of protein-coding sequences. Mol Biol Evol. 2006;23(1):7–9. 16177232 10.1093/molbev/msj021

[43] Nelson MI, Simonsen L, Viboud C, Miller MA, Taylor J, George KS, et al Stochastic processes are key determinants of short-term evolution in influenza A virus. Plos Pathogens. 2006;2(12):1144–51. 10.1371/journal.ppat.0020125 PMC1665651 17140286

[44] Paradis E, Claude J, Strimmer K. APE: Analyses of Phylogenetics and Evolution in R language. Bioinformatics. 2004;20(2):289–90. 14734327 10.1093/bioinformatics/btg412

[45] Bureau of Transportation Statistics. Air Carrier Statistics (Form 41 Traffic): T-100 Market (All Carriers). [Accessed 2013 June]; http://www.transtats.bts.gov/DL_SelectFields.asp?Table_ID=292.

[46] Centers for Disease Control and Prevention. Seasonal Influenza Q&A. 2013 [Accessed 2014 May]; http://www.cdc.gov/flu/about/qa/disease.htm.

[47] United States Census Bureau. 2000 County-to-County Worker Flow Files. 2000 [Accessed 2013 September]; http://www.census.gov/population/www/cen2000/commuting/index.html.

[48] Reichardt J, Bornholdt S. Statistical mechanics of community detection. Physical review E, Statistical, nonlinear, and soft matter physics. 2006;74(1):016110 16907154 10.1103/PhysRevE.74.016110

[49] Newman ME, Girvan M. Finding and evaluating community structure in networks. Physical review E, Statistical, nonlinear, and soft matter physics. 2004;69(2):026113 14995526 10.1103/PhysRevE.69.026113

[50] Traag VA, Bruggeman J. Community detection in networks with positive and negative links. Physical review E, Statistical, nonlinear, and soft matter physics. 2009;80(3):036115 19905188 10.1103/PhysRevE.80.036115

[51] Legendre P, Legendre L. Numerical Ecology. Amsterdam: Elsevier; 1998.

