# Supplementary material for: The Role of Human Transportation Networks in Mediating the Genetic Structure of Seasonal Influenza in the United States
Source: PLoS Pathog. 2015 Jun 18;11(6):e1004898. doi: 10.1371/journal.ppat.1004898 (PMC4472840; doi:10.1371/journal.ppat.1004898)
Supplement: S2 Table — Numbers in parentheses indicate the total number of publicly available sequences for those seasons; because of the extremely large sample size as compared to other seasons, subsamples were taken from states in these seasons that contributed an excessive number of sequences. (DOCX) [file ppat.1004898.s002.docx]

**Supplementary Tables and Figures**

|  | **H3N2** | | **H1N1** | |
| --- | --- | --- | --- | --- |
| **Season** | **Sequences (Full)** | **Locations** | **Sequences (Full)** | **Locations** |
| 2003-2004 | 191 | 29 | - | - |
| 2004-2005 | 189 | 34 | - | - |
| 2005-2006 | 147 | 30 | - | - |
| 2006-2007 | 211 | 34 | 371 | 28 |
| 2007-2008 | 662 (760) | 38 | 165 | 34 |
| 2008-2009 | 302 | 32 | 196 | 16 |
| 2010-2011 | 410 | 49 | 247 | 48 |
| 2011-2012 | 387 | 49 | 216 | 44 |
| 2012-2013 | 564 (1276) | 49 | 171 | 39 |

**Table S2.** Number of sequences per season and number of locations (US states) represented for influenza A/H3N2 and A/H1N1. Numbers in parentheses indicate the total number of publicly available sequences for those seasons; because of the extremely large sample size as compared to other seasons, subsamples were taken from states in these seasons that contributed an excessive number of sequences.
