# Supplementary material for: The Role of Human Transportation Networks in Mediating the Genetic Structure of Seasonal Influenza in the United States
Source: PLoS Pathog. 2015 Jun 18;11(6):e1004898. doi: 10.1371/journal.ppat.1004898 (PMC4472840; doi:10.1371/journal.ppat.1004898)
Supplement: S3 Table — ‘Root Height’ is measured in years before present, with the present time equal to the latest sampling date. ‘Clock rate’ is measured in substitutions/site/year. ‘Sequences’ represents the number of sequences analyzed per clade and ‘Locations’ represents the number of states these sequences were collected from. (DOCX) [file ppat.1004898.s003.docx]

| **Season** | **Root Height** | **Growth Rate** | **Clock Rate** | **Clade** | **Clade Divergence Date** | **Sequences** | **Locations** |
| --- | --- | --- | --- | --- | --- | --- | --- |
| 2003-2004 | 1.424  (1.02-1.909) | 3.669  (1.87-5.555) | 0.005572  (0.003461-0.007759) | 1 | 2003.598 (2003.424 - 2003.738) | 30 | 12 |
|  |  |  |  | 2 | 2003.282 (2002.925 - 2003.581) | 59 | 17 |
| 2004-2005 | 1.656  (1.087-2.278) | 3.764  (2.005-5.592) | 0.004694  (0.003013-0.006469) | NA | NA | NA | NA |
| 2005-2006 | 1.35  (0.951-1.816) | 2.636  (1.243-4.181) | 0.009151  (0.006521-0.02082) | 1 | 2005.337 (2005.066 - 2005.572) | 49 | 17 |
|  |  |  |  | 2 | 2005.784 (2005.662 - 2005.886) | 29 | 10 |
| 2006-2007 | 1.647  (1.137-2.251) | 2.713  (1.306-4.2) | 0.007733  (0.005568-0.009923) | 1 | 2006.684 (2006.533 - 2006.819) | 50 | 15 |
|  |  |  |  | 2 | 2006.684 (2006.533 - 2006.819) | 112 | 29 |
| 2007-2008 | 1.528  (1.165-1.962) | 3.391  (2.142-4.782) | 0.006833  (0.005487-0.008296) | 1 | 2007.344 (2007.223 - 2007.578) | 78 | 29 |
|  |  |  |  | 2 | 2007.752 (2007.613 - 2007.876) | 28 | 16 |
|  |  |  |  | 3 | 2007.752 (2007.613 - 2007.876) | 159 | 32 |
|  |  |  |  | 4 | 2007.679 (2007.536 - 2007.811) | 107 | 25 |
|  |  |  |  | 5 | 2007.679 (2007.536 - 2007.811) | 277 | 32 |
| 2008-2009 | 1.532  (1.007-2.073) | 2.084  (1.163-2.964) | 0.006681  (0.005259-0.008227) | NA | NA | NA | NA |
| 2010-2011 | 1.947  (1.488-2.42) | 3.486  (2.362-4.683) | 0.005383  (0.004137-0.006641) | 1 | 2010.523 (2010.320 - 2010.709) | 23 | 16 |
|  |  |  |  | 2 | 2010.566 (2010.401 - 2010.731) | 62 | 23 |
| 2011-2012 | 2.205  (1.723-2.741) | 2.697  (1.906-3.531) | 0.00577  (0.0041006-0.0062034) | 1 | 2011.397 (2011.773-2011.365) | 41 | 21 |
|  |  |  |  | 2 | 2011.191 (2011.577-2011.14) | 67 | 23 |
|  |  |  |  | 3 | 2011.301 (2011.69-2011.263) | 49 | 26 |
|  |  |  |  | 4 | 2011.805 (2011.897-2011.65) | 23 | 9 |
| 2012-2013 | 2.841  (2.178-3.603) | 2.088  (1.347-2.864) | 0.004942  (0.003892-0.006008) | 1 | 2012.591 (2012.418 - 2012.746) | 21 | 11 |
|  |  |  |  | 2 | 2012.621 (2012.445 - 2012.774) | 20 | 9 |
|  |  |  |  | 3 | 2012.450 (2012.263 - 2012.618) | 33 | 22 |
|  |  |  |  | 4 | 2012.354 (2012.137 - 2012.561) | 35 | 15 |
|  |  |  |  | 5 | 2012.354 (2012.137 - 2012.561) | 37 | 19 |
|  |  |  |  | 6 | 2012.616 (2012.466 - 2012.750) | 46 | 23 |

**Table S3.** Summary of epidemiological and evolutionary dynamics of H3N2 epidemics based on phylogenetic analyses of each influenza season. ‘Root Height’ is measured in years before present, with the present time equal to the latest sampling date. ‘Clock rate’ is measured in substitutions/site/year. ‘Sequences’ represents the number of sequences analyzed per clade and ‘Locations’ represents the number of states these sequences were collected from.
