# Supplementary figures and images for: The Role of Human Transportation Networks in Mediating the Genetic Structure of Seasonal Influenza in the United States
Source: PLoS Pathog. 2015 Jun 18;11(6):e1004898. doi: 10.1371/journal.ppat.1004898 (PMC4472840; doi:10.1371/journal.ppat.1004898)

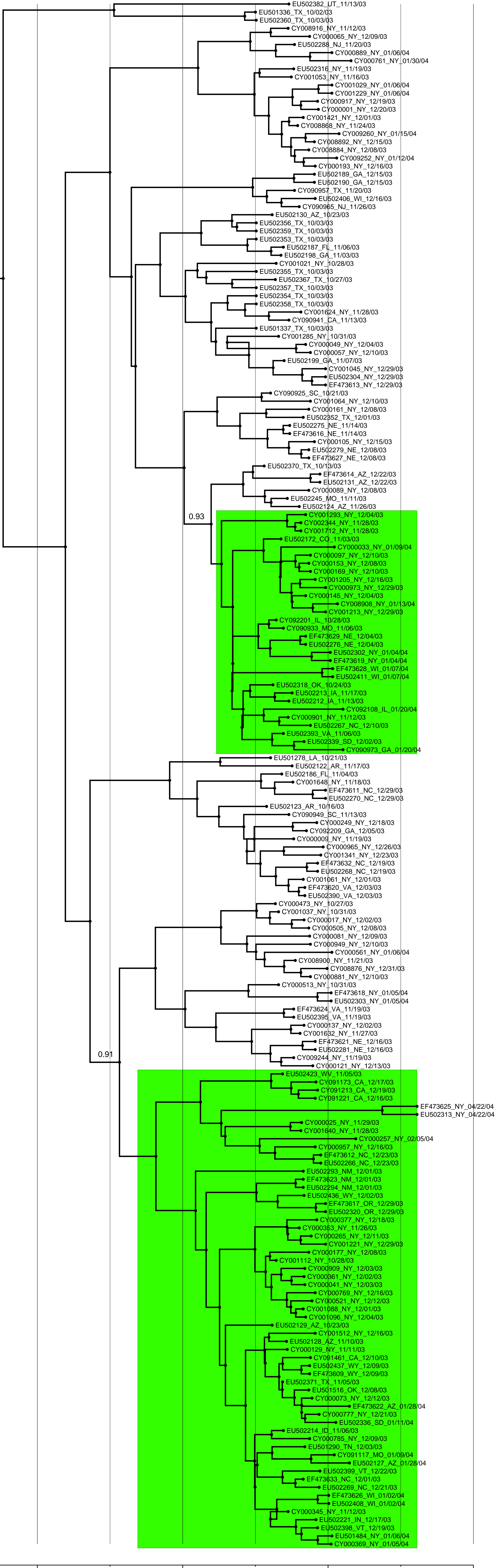

Supplement: S1 Fig — Clades used for association tests are highlighted in green. Posterior probability values (>0.9) are labeled for nodes leading to clades used in the correlation analysis. Horizontal axis is measured in years. (PDF) [file ppat.1004898.s005.pdf]

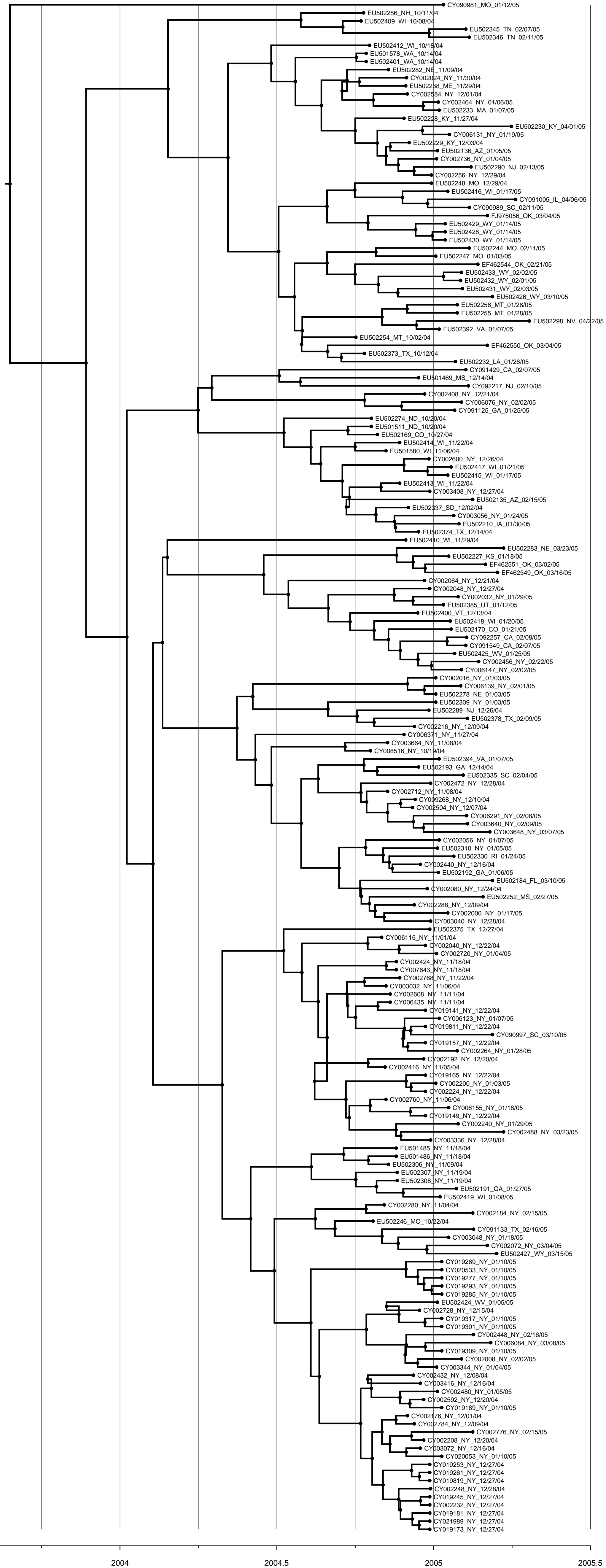

Supplement: S2 Fig — Horizontal axis is measured in years. (PDF) [file ppat.1004898.s006.pdf]

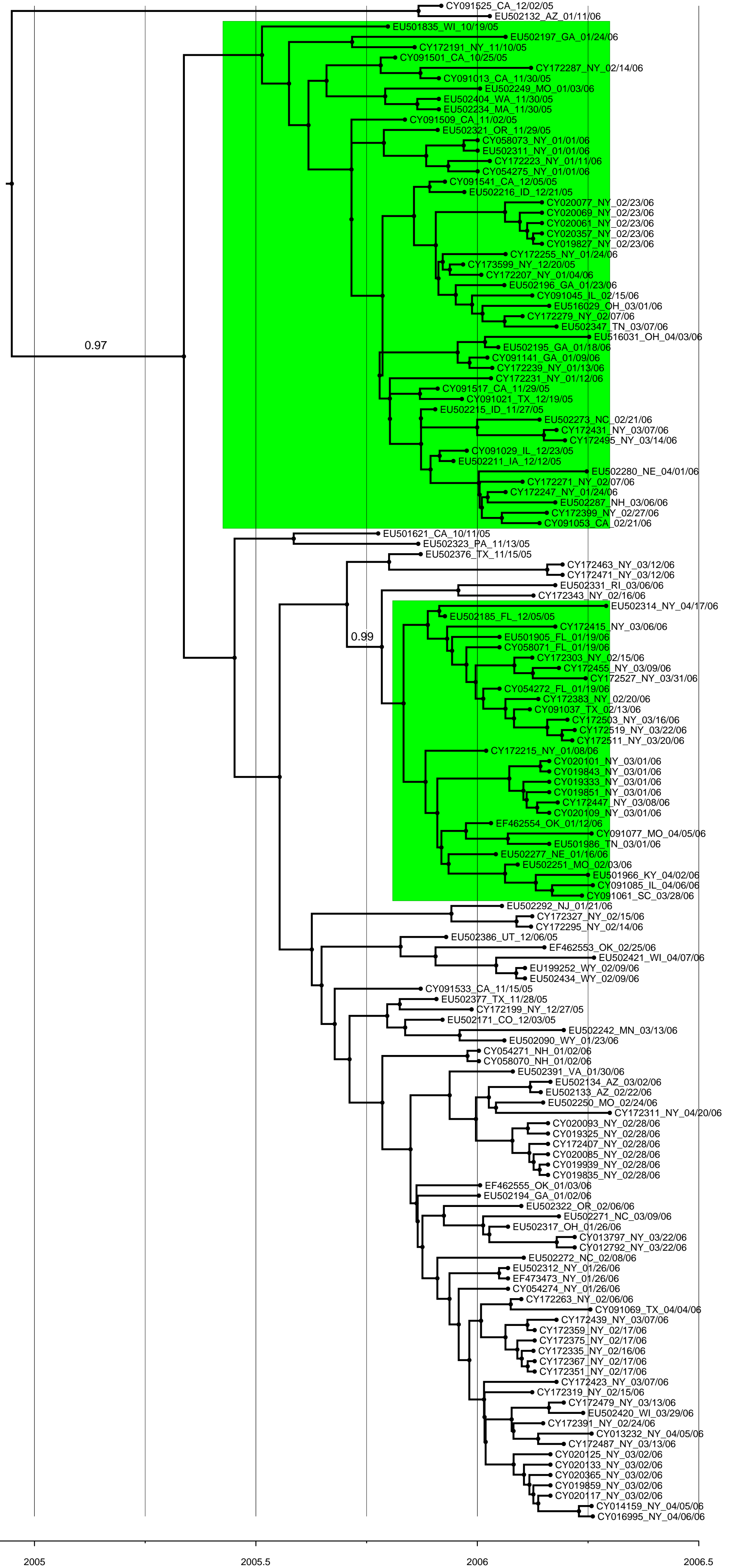

Supplement: S3 Fig — Clades used for association tests are highlighted in green. Posterior probability values (>0.9) are labeled for nodes leading to clades used in the correlation analysis. Horizontal axis is measured in years. (PDF) [file ppat.1004898.s007.pdf]

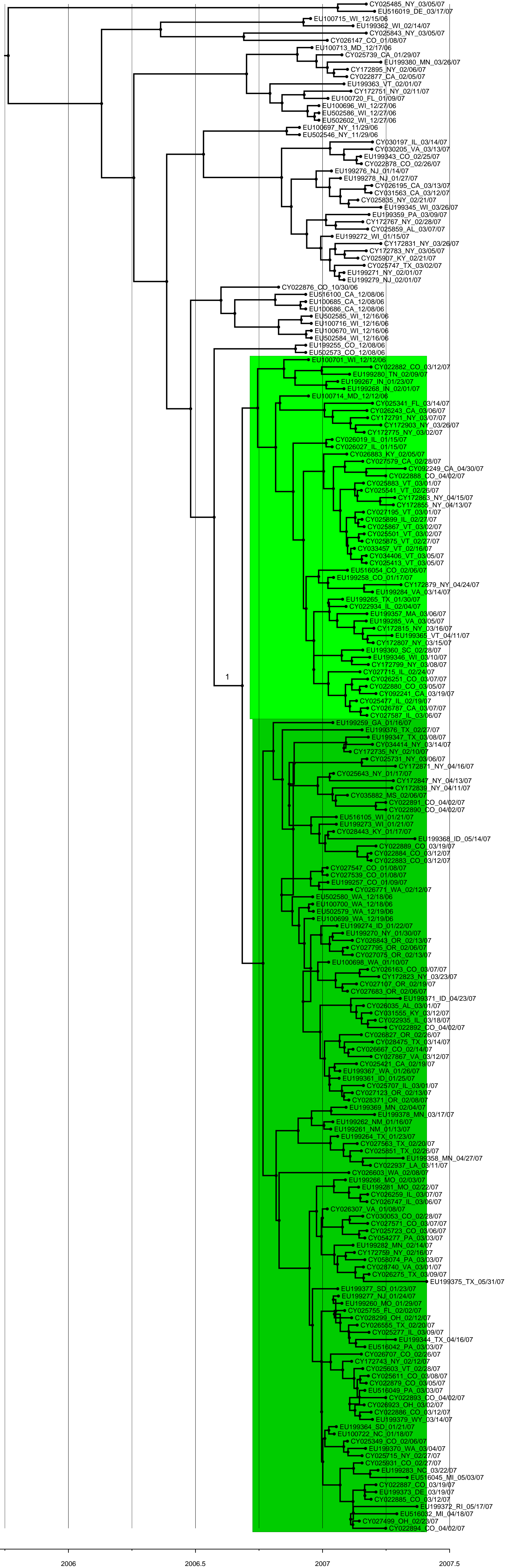

Supplement: S4 Fig — Clades used for association tests are highlighted in green. Posterior probability values (>0.9) are labeled for nodes leading to clades used in the correlation analysis. Horizontal axis is measured in years. (PDF) [file ppat.1004898.s008.pdf]

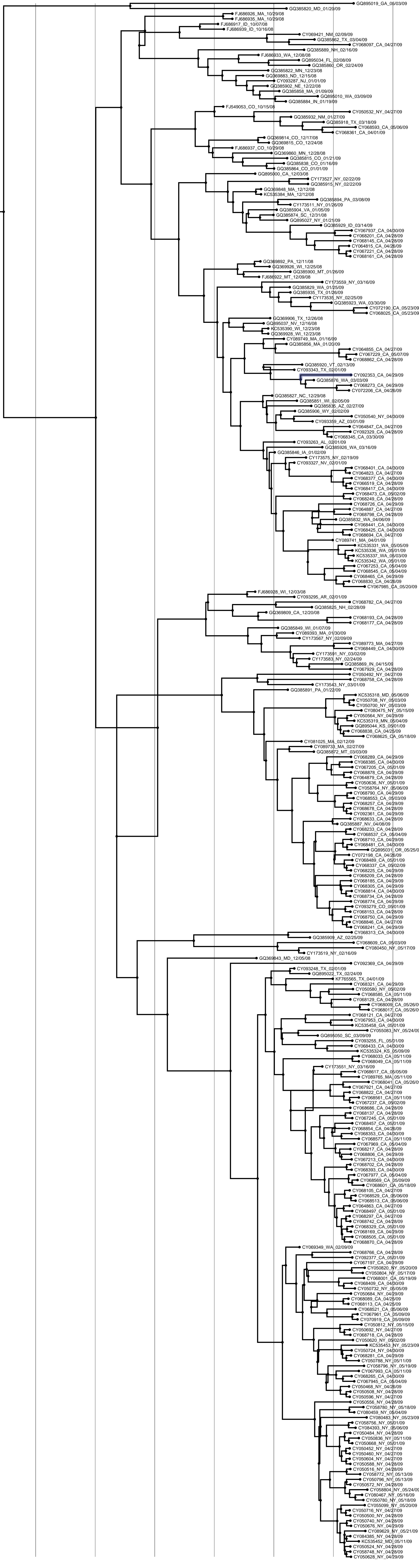

2008

2008.5

2009

2009.5

Supplement: S6 Fig — Horizontal axis is measured in years. (PDF) [file ppat.1004898.s010.pdf]

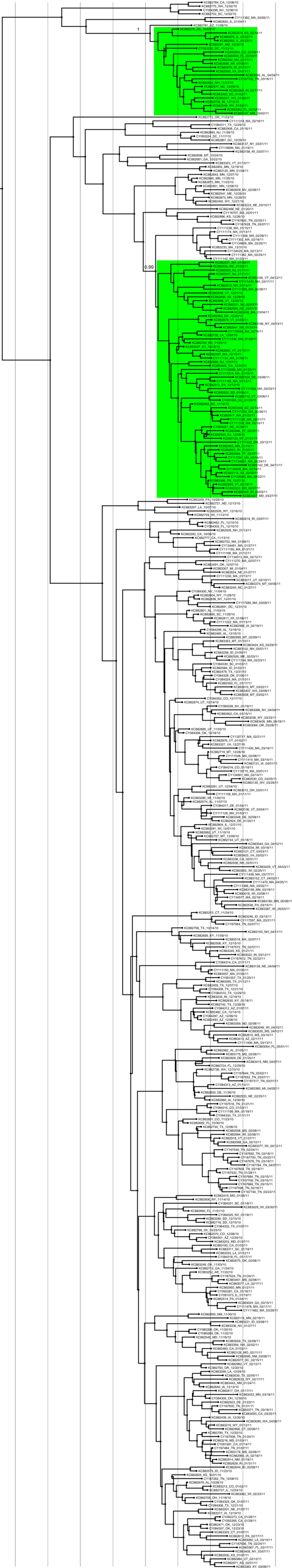

Supplement: S7 Fig — Clades used for association tests are highlighted in green. Posterior probability values (>0.9) are labeled for nodes leading to clades used in the correlation analysis. Horizontal axis is measured in years. (PDF) [file ppat.1004898.s011.pdf]

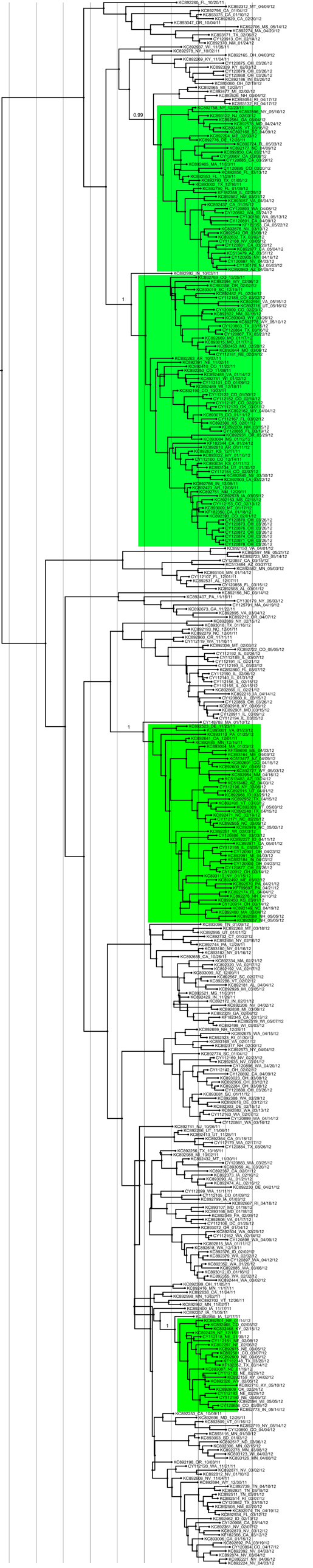

Supplement: S8 Fig — Clades used for association tests are highlighted in green. Posterior probability values (>0.9) are labeled for nodes leading to clades used in the correlation analysis. Horizontal axis is measured in years. (PDF) [file ppat.1004898.s012.pdf]

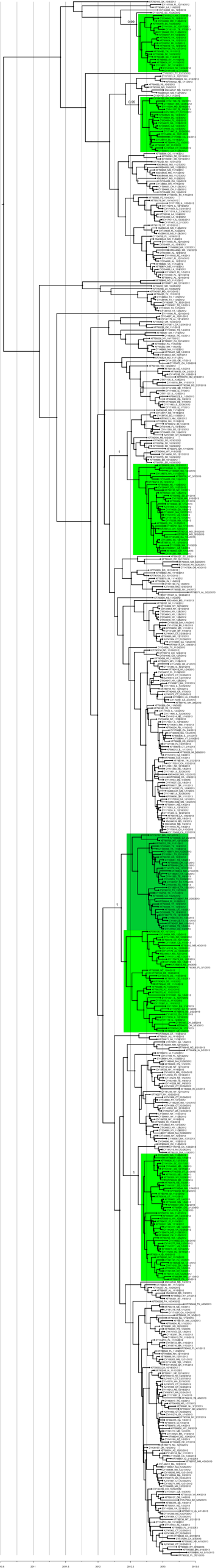

Supplement: S9 Fig — Clades used for association tests are highlighted in green. Posterior probability values (>0.9) are labeled for nodes leading to clades used in the correlation analysis. Horizontal axis is measured in years. (PDF) [file ppat.1004898.s013.pdf]

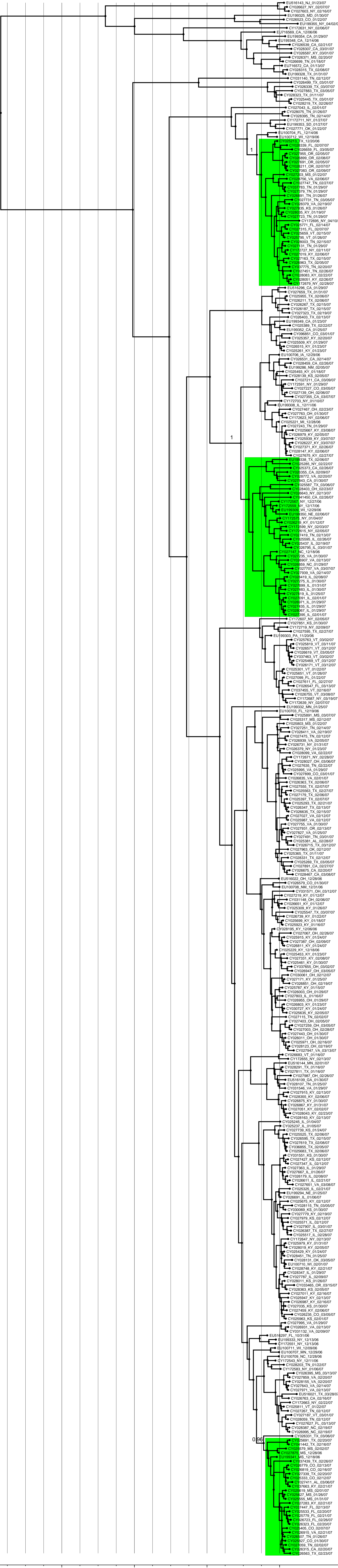

Supplement: S10 Fig — Clades used for association tests are highlighted in green. Posterior probability values (>0.9) are labeled for nodes leading to clades used in the correlation analysis. Horizontal axis is measured in years. (PDF) [file ppat.1004898.s014.pdf]

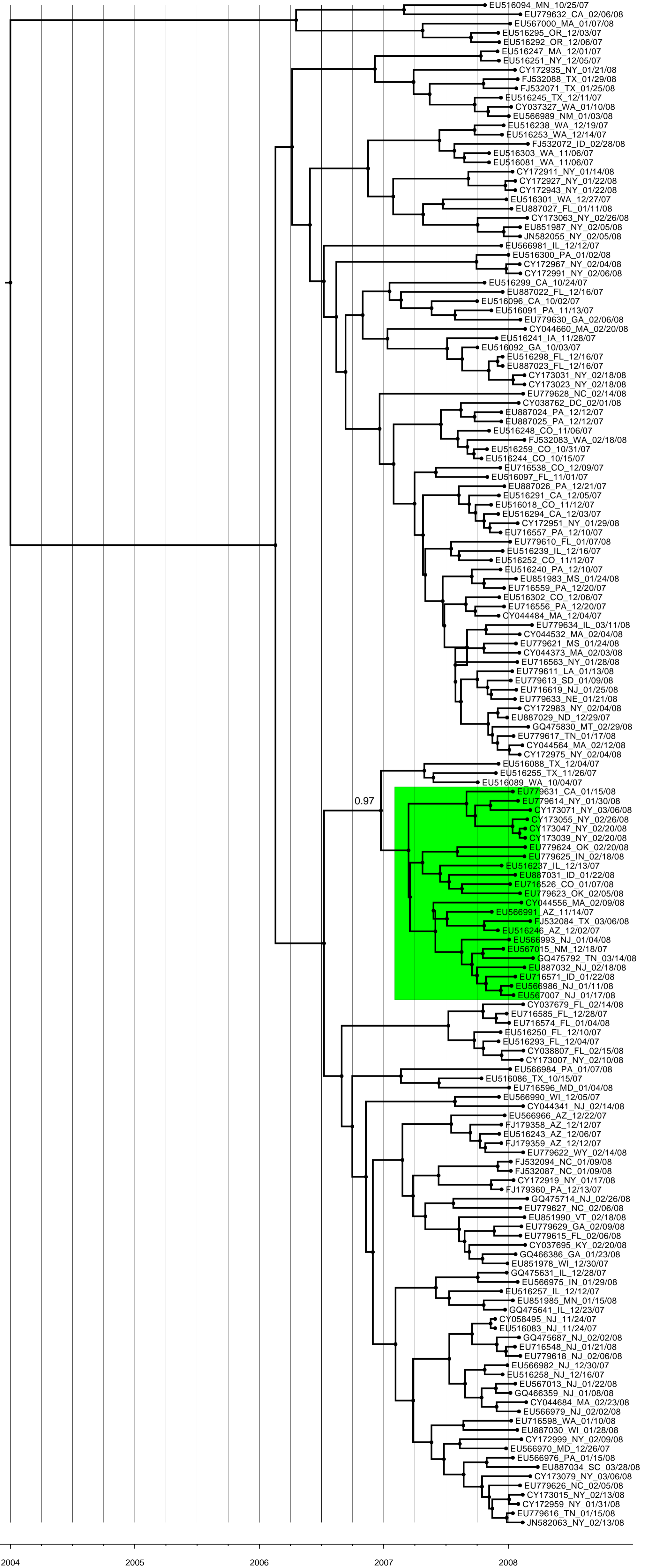

Supplement: S11 Fig — Clades used for association tests are highlighted in green. Posterior probability values (>0.9) are labeled for nodes leading to clades used in the correlation analysis. Horizontal axis is measured in years. (PDF) [file ppat.1004898.s015.pdf]

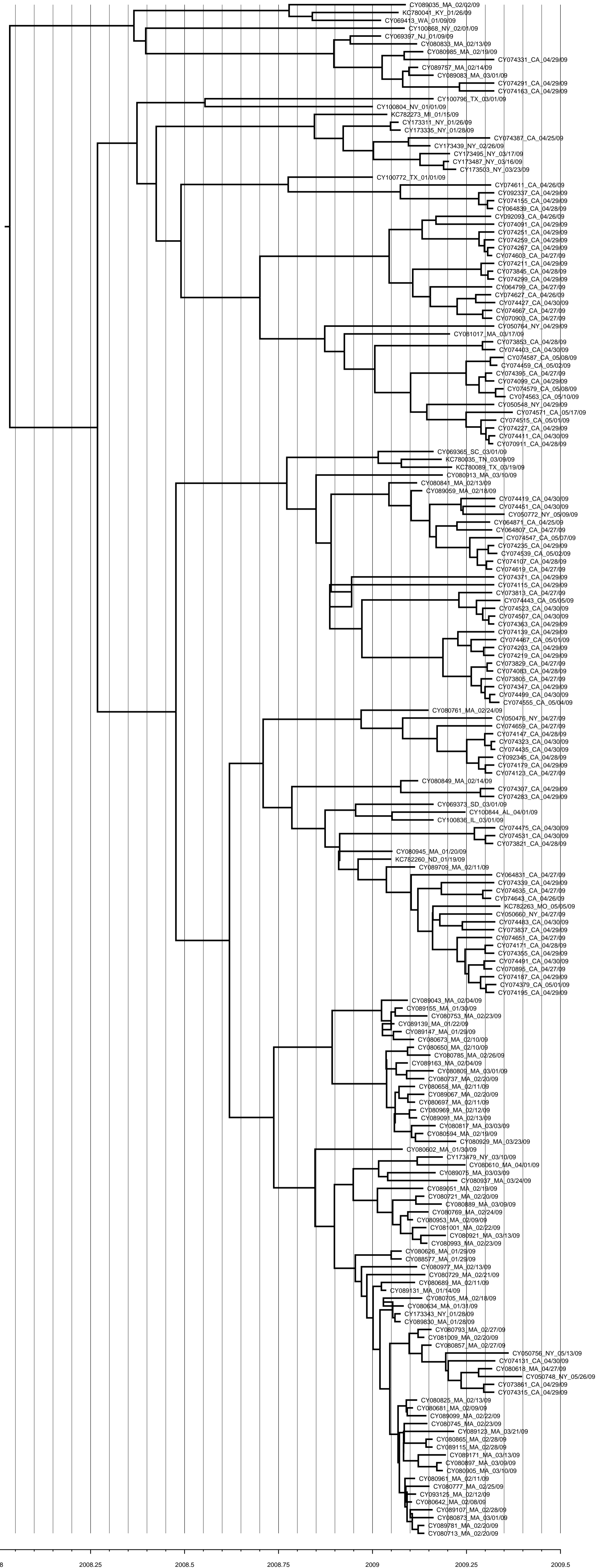

Supplement: S12 Fig — Horizontal axis is measured in years. (PDF) [file ppat.1004898.s016.pdf]

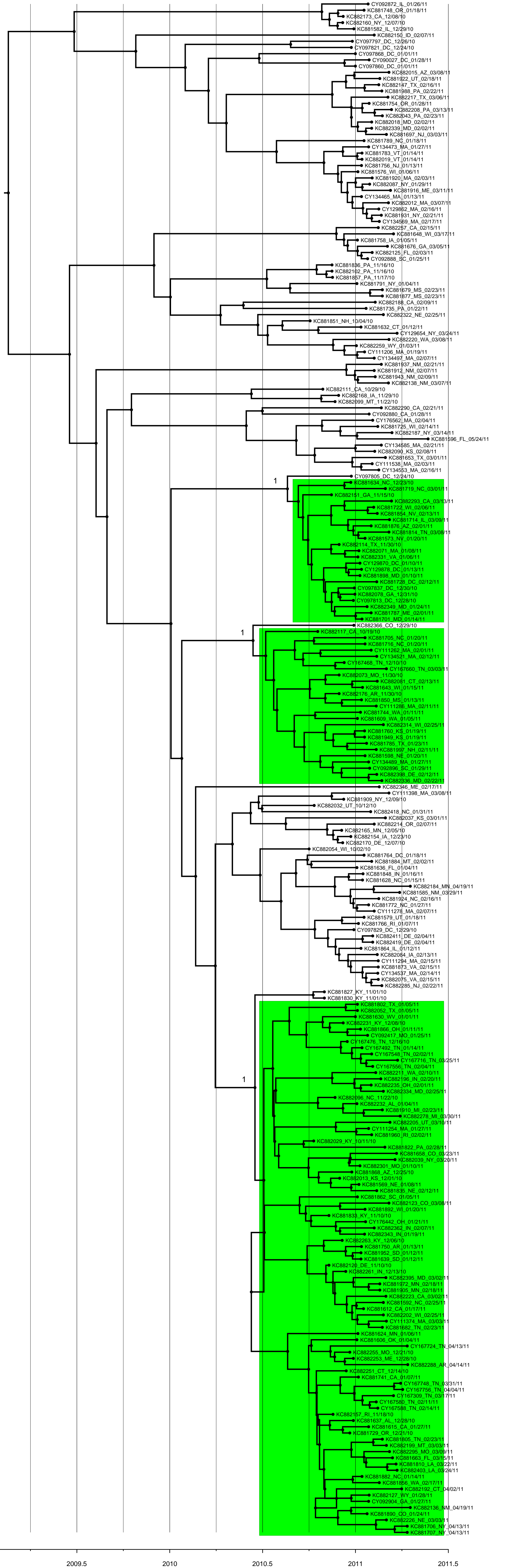

Supplement: S13 Fig — Clades used for association tests are highlighted in green. Posterior probability values (>0.9) are labeled for nodes leading to clades used in the correlation analysis. Horizontal axis is measured in years. (PDF) [file ppat.1004898.s017.pdf]

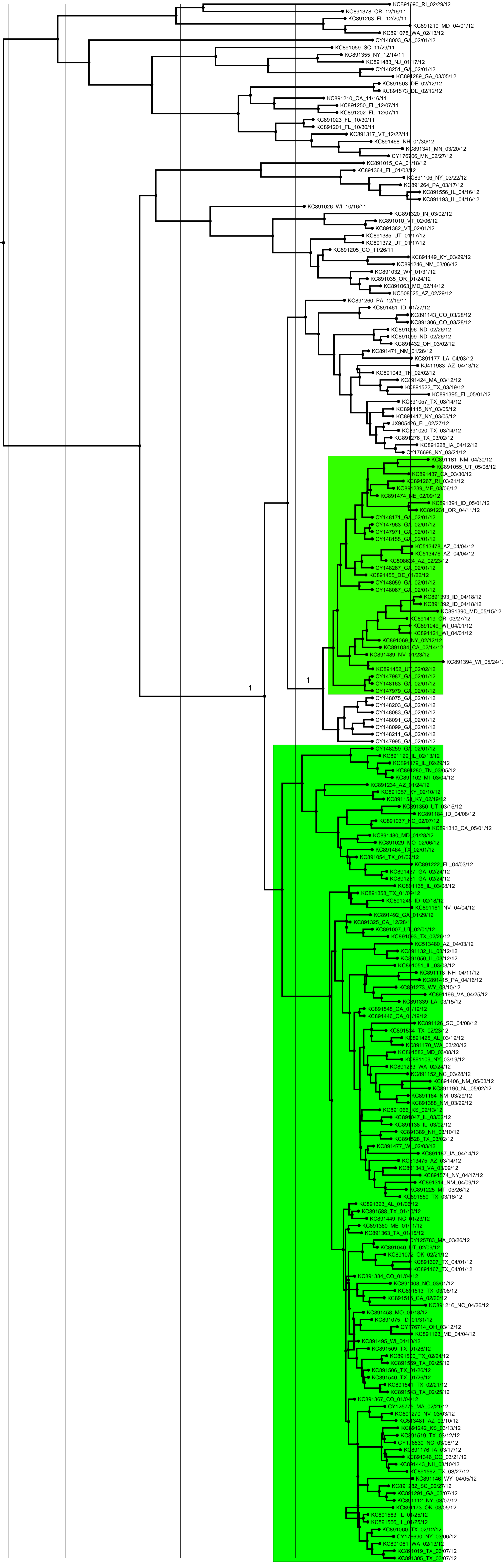

Supplement: S14 Fig — Clades used for association tests are highlighted in green. Posterior probability values (>0.9) are labeled for nodes leading to clades used in the correlation analysis. Horizontal axis is measured in years. (PDF) [file ppat.1004898.s018.pdf]

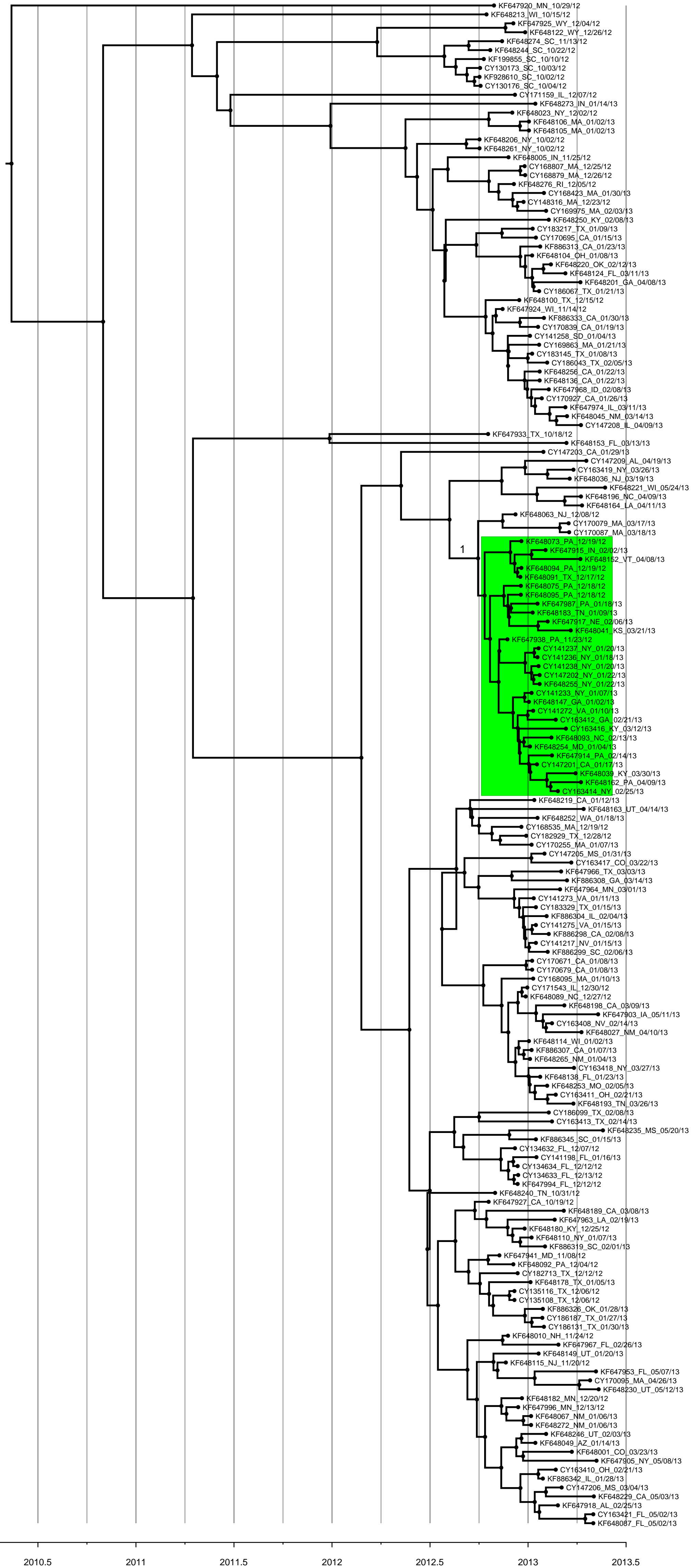

Supplement: S15 Fig — Clades used for association tests are highlighted in green. Posterior probability values (>0.9) are labeled for nodes leading to clades used in the correlation analysis. Horizontal axis is measured in years. (PDF) [file ppat.1004898.s019.pdf]
