# Supplementary material for: The Role of Human Transportation Networks in Mediating the Genetic Structure of Seasonal Influenza in the United States
Source: PLoS Pathog. 2015 Jun 18;11(6):e1004898. doi: 10.1371/journal.ppat.1004898 (PMC4472840; doi:10.1371/journal.ppat.1004898)
Supplement: S5 Fig — Clades used for association tests are highlighted in green. Posterior probability values (>0.9) are labeled for nodes leading to clades used in the correlation analysis. Horizontal axis is measured in years. (PDF) [file ppat.1004898.s009.pdf]

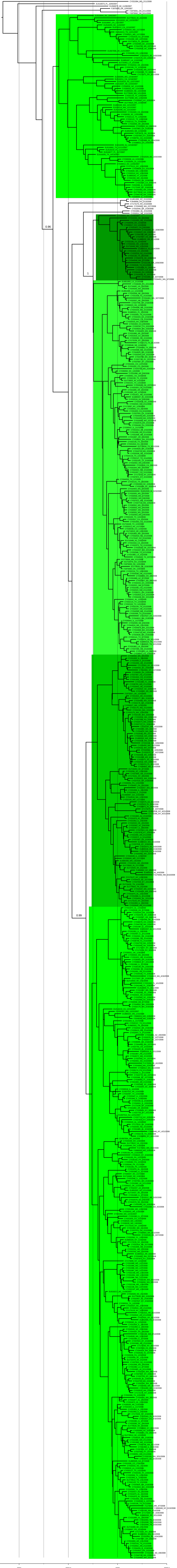
0.96
1
0.99
2007
2007.5
2008
2008.5
